# Alcohol management plans in Aboriginal and Torres Strait Islander (Indigenous) Australian communities in Queensland: community residents have experienced favourable impacts but also suffered unfavourable ones

**DOI:** 10.1186/s12889-016-3995-8

**Published:** 2017-01-10

**Authors:** Alan R. Clough, Stephen A. Margolis, Adrian Miller, Anthony Shakeshaft, Christopher M. Doran, Robyn McDermott, Robert Sanson-Fisher, Valmae Ypinazar, David Martin, Jan A. Robertson, Michelle S. Fitts, Katrina Bird, Bronwyn Honorato, Simon Towle, Caryn West

**Affiliations:** 1 Community-based Health Promotion and Prevention Studies Group, Australian Institute of Tropical Health and Medicine, James Cook University, Cairns, QLD 4870 Australia; 2 School of Medicine, Griffith University, Nathan, QLD 4111 Australia; 3 Indigenous Research Unit, Griffith University, Nathan, QLD 4111 Australia; 4 National Drug and Alcohol Research Centre, University of New South Wales, Sydney, NSW 2052 Australia; 5 Health Economics, Central Queensland University, Brisbane, 4000 QLD Australia; 6 Centre for Chronic Disease Prevention, Australian Institute of Tropical Health and Medicine, College of Public Health, Medical and Veterinary Sciences, James Cook University, Cairns, QLD 4870 Australia; 7 School of Medicine and Public Health (Public Health), University of Newcastle, Sydney, NSW 2000 Australia; 8 College of Arts and Social Sciences, Australian National University, Canberra, Australia; 9 Division of Tropical Health and Medicine, College of Public Health, Medical & Veterinary Sciences, James Cook University, Cairns, QLD Australia; 10 Division of Tropical Health and Medicine, College of Healthcare Sciences, James Cook University, Cairns, QLD Australia

**Keywords:** Alcohol, Indigenous, Aboriginal and Torres Strait Islander, Australian, Legal intervention, Evaluation

## Abstract

**Background:**

In Australia, ‘Alcohol Management Plans’ (AMPs) provide the policy infrastructure for State and Commonwealth Governments to address problematic alcohol use among Aboriginal and Torres Strait Islanders. We report community residents’ experiences of AMPs in 10 of Queensland’s 15 remote Indigenous communities.

**Methods:**

This cross-sectional study used a two-stage sampling strategy: *N* = 1211; 588 (48%) males, 623 (52%) females aged ≥18 years in 10 communities. Seven propositions about ‘favourable’ impacts and seven about ‘unfavourable’ impacts were developed from semi-structured interviews. For each proposition, one-sample tests of proportions examined participant agreement and multivariable binary logistic regressions assessed influences of gender, age (18–24, 25–44, 45–64, ≥65 years), residence (≥6 years), current drinking and Indigenous status. Confirmatory factor analyses estimated scale reliability (*ρ*), item loadings and covariances.

**Results:**

Slim majorities agreed that: AMPs reduced violence (53%, *p* = 0.024); community a better place to live (54%, 0.012); and children were safer (56%, *p* < 0.001). More agreed that: school attendance improved (66%, *p* < 0.001); and awareness of alcohol’s harms increased (71%, *p* < 0.001). Participants were equivocal about improved personal safety (53%, *p* = 0.097) and reduced violence against women (49%, *p* = 0.362). The seven ‘favourable’ items reliably summarized participants’ experiences of reduced violence and improved community amenity (*ρ* = 0.90).

Stronger agreement was found for six ‘unfavourable’ items: alcohol availability not reduced (58%, *p* < 0.001); drinking not reduced (56%, *p* < 0.001)); cannabis use increased (69%, *p* < 0.001); more binge drinking (73%, *p* < 0.001); discrimination experienced (77%, *p* < 0.001); increased fines, convictions and criminal records for breaching restrictions (90%, *p* < 0.001). Participants were equivocal (51% agreed, *p* = 0.365) that police could enforce restrictions effectively. ‘Unfavourable’ items were not reliably reflected in one group (*ρ* = 0.48) but in: i) alcohol availability and consumption not reduced and ii) criminalization and discrimination.

In logistic regressions, longer-term (≥ 6 years) residents more likely agreed that violence against women had reduced and that personal safety had improved but also that criminalization and binge drinking had increased. Younger people disagreed that their community was a better place to live and strongly agreed about discrimination. Current drinkers’ views differed little from the sample overall.

**Conclusions:**

The present Government review provides an opportunity to reinforce ‘favourable’ outcomes while targeting: illicit alcohol, treatment and diversion services and reconciliation of criminalization and discrimination issues.

**Electronic supplementary material:**

The online version of this article (doi:10.1186/s12889-016-3995-8) contains supplementary material, which is available to authorized users.

## Background

For Indigenous populations living in remote parts of the western United States [1, 2] and Alaska [3–8], northern Canada [9, 10], Greenland [11], and in rural [12–14] and remote [15–21] Australia, robust legal and regulatory interventions to address alcohol misuse and associated harms have been shown to have mainly positive effects. In Australia, for Indigenous (Aboriginal and Torres Strait Islander) populations living in remote areas, regulatory interventions known as ‘Alcohol Management Plans’ (AMPs) have been used by State and Commonwealth Governments during the past two decades [22, 23].

In the State of Queensland, its Government first implemented AMPs in 2002 [24], explicitly to protect women and children and to reduce very high rates of injury and death documented during the latter decades of the 20th century [25, 26]. Nineteen Indigenous communities were designated as among the state’s most vulnerable in a 2001 Government-commissioned inquiry [26]. In response to its inquiry, and to strong advocacy for action on what was widely seen as a public health emergency [27], between 2002 and 2006, the Government declared all 19 communities (situated in 15 Local Government Areas) as ‘restricted areas’ where permitted quantities and types of alcohol are determined by Government [23].

Prior to 2002, from the 1980s, alcohol had been available (for on-premises consumption) from outlets operated and managed under licences held by the elected Indigenous Local Government Councils [22, 23]. Additionally, takeaway alcohol was readily available with few specific restrictions from these and from other liquor outlets in the neighbouring rural towns and regional centres [23]. AMPs first imposed strict penalties for the possession and consumption of prescribed quantities and types of liquor within a community’s ‘restricted area’ boundary, but with appropriate exemptions for the continued operation of the community-managed liquor outlets [23]. Subsequent Government reviews conducted during 2005 to 2007 [28] brought even more stringent controls and stronger punitive measures. By the end of 2008, the licences of most of the community-run liquor outlets were terminated or modified, effectively prohibiting all alcohol in seven communities [23]. Police powers to search for and seize illicit alcohol were increased by the Queensland Government, and liquor outlets in the neighbouring towns within the very large, Government-defined ‘catchments’ of AMP communities became subject to ‘minimising harm’ provisions to prevent the sale of prohibited alcohol that could reach the ‘restricted areas’ [23].

Then, at the end of 2011, with few options left to further tighten restrictions, a decade after AMPs were first introduced and with an abrupt about-face, successive Queensland Governments began to seek ‘exit strategies’ from these long-standing alcohol controls, promising to review AMPs as part of their policy platforms for the Queensland electorate [29]. For its review, consistent with its determination to control policy decisions about AMPs, the Government requires the Local Government Councils to consult with their constituent populations to prepare and submit proposals for subsequent consultation and final Government determination [29].

Throughout all this, the views and experiences of the usual residents of the affected communities have never been documented and their priorities for alcohol controls never considered. In a related publication, we have already reported the views of a large sample of key service providers and community leaders [15]. These key people attributed to AMPs an abrupt reduction in violence with improved community amenity, particularly for the more remote communities with prohibition. However, they also suggested that over time the availability of illicit alcohol and an urgency to consume it, migration to larger centres to seek alcohol, criminalization, substitution of illicit drugs for alcohol, changed drinking behaviours and discrimination had eroded these achievements [15]. Recognising that the usual residents of the affected communities have neither had the opportunity to provide their views on these matters, nor have they ever received any objectively-derived evidence that AMPs had in fact reduced violence and injury and improved the safety of women and children, we aimed to document community peoples’ experiences and perceptions of the impacts of AMPs at the local level. We sought to better understand ‘favourable’ impacts achieved by AMPs while identifying any unintended, ‘unfavourable’ ones that should be addressed.

## Methods

### Communities, participants and data

The affected populations and the complex policy and regulatory underpinnings of Queensland’s AMPs have already been described in detail [19, 23, 26]. In sum, Queensland’s strategies to control alcohol in Aboriginal and Torres Strait Islander communities have featured ever-more-intense and complex techniques to restrict alcohol availability, until recently. In official statistics in Australia, ‘very remote’ or ‘remote’ Indigenous communities typically comprise small, discrete clusters of people and dwellings with from 400 to 3500 people [30]. At the 2011 census, just one quarter of Australia’s Indigenous population of 670,000 lived in these kinds of communities [31]. In 2011, a total of 17,485 Indigenous and non-Indigenous people resided in the 19 communities (15 restricted areas) with an AMP in place [32]. This study was conducted in 10 of these during 2014–15. Combined, the 10 study communities had a total resident population of 9480, including 5989 adults (aged 18 years and over, the legal drinking age in Australia) of whom 92% (5497) were Indigenous.

English is widely spoken in these localities, although it is often a second language (or a creole) with skills in English communication varying widely; bringing specific challenges for survey work. Many Indigenous residents of AMP communities have lived much of their lives there and many non-Indigenous residents have also lived in one or more AMP communities for long periods. Traditional connections to culture, extended families, to land and sea country [33–35] remain very strong. Some AMP communities are situated on islands near the coast, but the 10 communities surveyed are all on the mainland. Road journeys to the nearest liquor outlets, located on connecting roads and highways and in regional towns and centres in ‘catchment’ areas, vary from 3 km up to around 350 km. Vehicle access has mostly been via unsealed roads, until recently, with the quality of sealed roads significantly improved over the past decade. Although some communities can still be isolated for several months during the tropical wet season, there are 29 liquor outlets in their ‘catchment’ areas which have ‘minimizing harm’ conditions on their licences specifically designed to limit alcohol supply to residents of the neighbouring restricted areas [23].

### Selection of communities and participants

The 10 communities surveyed were all those where the Indigenous Local Government Council invited the research team to do the work. A simple random sample with around 800 of the adult residents could detect a minimum difference of +/− 5% in the proportion in the sample agreeing with a survey item compared with a reference value of 50% (representing evenly divided opinion) with adequate study power (~0.80). Sampling was multi-stage with ‘remote’ (six communities) or ‘very remote’ (four communities) being the first stage and the community level the second stage. In previous substance use surveys conducted in some of the 10 communities [36–38], despite their small populations, sample sizes of at least 100 participants in similar age groups in a community have been feasible. With this sampling goal in mind for each community, the total sample size was increased by 5% to allow for missing data, 20% to control for confounding in regressions and 20% for the design effect, making a final planned sample size of 1160 [39].

Random selection of participants was not an option given that a key objective was to provide all residents the opportunity to participate and share their views and experiences. Following strategies used in similar studies in these communities, the survey was advertised through local communication channels several weeks prior. Then, participants were recruited opportunistically throughout each community, usually in public places such as at the entrances to the community grocery store, community businesses and service centres, the community health clinic, at sporting or community events and in workplaces. Project staff travelled from the study’s headquarters in the major regional centre (Cairns) in far north Queensland, residing in the communities for from 1 to 3 weeks to conduct the surveys. Indigenous cultural brokers, employed to explain the survey objectives also assisted with recruiting and consenting participants. Strong views for and against AMPs have been vigorously defended in the communities in the past, and there is long-standing suspicion within these marginalized populations of Government and other external agents. To maintain transparency and to ensure project staff and participants felt safe, all surveys were conducted in public places with no interviews conducted in private unless specific requests were made. Most participants completed the survey in approximately 20 min.

### Survey design

In an already-published study, we defined ‘favourable’ and ‘unfavourable’ impacts using semi-structured interviews conducted with a large sample of key community leaders, service providers and other key stakeholders who have a mandate for managing alcohol-related issues and consequences of AMPs in rural and remote Queensland [15]. The present study used these definitions to develop a draft survey to document, for the first time, the views and experiences of community people who are directly affected by AMPs. The draft survey was developed from the first 83 of these semi-structured interviews with key people [15]. Two lines of questioning imposed a thematic structure on the semi-structured interview information:

- What are the ‘favourable’ things that the AMPs have achieved?
- What are the ‘unfavourable’ things that have happened because of AMPs?

Key sub-themes within each theme were turned into propositions for the survey. The procedures for coding themes and sub-themes in the semi-structured interview information and testing coder agreement have already been described in full [15]. In summary, three project officers coded the information from interviews to candidate sub-themes. The coders and members of the interview team then reviewed the coded sub-themes and, by consensus, developed preliminary sets of propositions about ‘favourable’ and ‘unfavourable’ impacts to be included in the draft survey.

#### Propositions about perceived ‘favourable’ impacts

Reductions in levels of violence generally and in vulnerable groups in particular (women and children) were reported along with improved community safety and amenity overall. Some of the 83 participants interviewed believed that greater awareness of alcohol’s harms came after AMPs were implemented. Improved conditions for children were believed to be reflected in increased school attendance [15]. The set of seven ‘favourable’ propositions listed in Table 1 was developed from these sub-themes.

**Table 1:** Proportions of participants agreeing with seven ‘favourable’ propositions and seven ‘unfavourable’ propositions about possible impacts of Alcohol Management Plans (AMPs) put to 1211 residents of 10 Aboriginal and Torres Strait Islander (Indigenous) communities in a survey conducted in Queensland (Australia) in 2014–15

Variable name	Propositions (to avoid conditioned responses propositions were arranged in the survey according to the order specified by the number enclosed in brackets)	Proportion of participants who ‘agree’ (*n* responding)	p*
‘Favourable’ impacts			
f1	The AMP has helped make children safer in this community (6)	56% (1007)	<0.001
f2	The AMP has made people more safe in this community (11)	53% (1019)	0.097
f3	The AMP has reduced violence against women in this community (4)	49% (1017)	0.363
f4	Since the AMP, violence has gone down in this community (5)	53% (1072)	0.024
f5	Since the AMP, school attendance has gone up in this community (7)	66% (899)	<0.001
f6	The AMP has been good for this community and made it a better place to live (1)	54% (1026)	0.012
f7	People are more aware of harmful effects of alcohol/drinking now (since the AMP) (2)	71% (1057)	<0.001
‘Unfavourable’ impacts			
u4	The AMP has caused more people to get fined, criminal records and convictions (3)	90% (1064)	<0.001
u1	There is more (not so much) gunjah being smoked in this community since the AMP (12) †	69% (944)	<0.001
u3	There is more “binge drinking” now than before the AMP (13)	73% (1006)	<0.001
u6	The AMP has discriminated against some people (14)	77% (1026)	<0.001
u5	Police can’t (can) enforce the AMP effectively and stop the alcohol coming in (9) †	51% (1098)	0.365
u7	The AMP has not (has) reduced the alcohol people can get in this community (8) †	58% (1118)	<0.001
u8	The AMP has not (has) helped people change their drinking and they are (not) drinking less (10) †	56% (1076)	<0.001

* One-sample test of proportions - stated proportion agreeing is different from a theoretical reference proportion of 50%, i.e. no majority agreeing/disagreeing

† These propositions were put to participants with reverse logic but then reverse coded for analysis to reduce possible bias where participants’ views may have been led towards agreeing with some of the more critical and contentious unfavourable impacts

#### Propositions about perceived ‘unfavourable’ impacts

‘Unfavourable’ impacts were reflected in concerns among the 83 interviewed about continued access to illicit alcohol, with ‘binge drinking’ seen to be common. There was a widespread belief that police had not the resources to enforce restrictions to the full extent demanded by legislation. Increased criminalization from prosecutions for breaching restrictions was a concern for some interviewed, along with the discrimination observed and experienced as restrictions have been seen to impact Indigenous community residents selectively with little or no impact on the residents of neighbouring towns or, indeed, any other populations in Queensland [15]. The substitution of mood altering (illicit) drugs, particularly cannabis, for alcohol was also linked with alcohol restrictions in the minds of those interviewed (reported in detail in a separate publication). A second preliminary set of seven ‘unfavourable’ propositions was developed from this evidence (Table 1).

The suitability and applicability of the draft survey was examined and the wording for the propositions discussed in detail with elected Indigenous Local Government Councilors, community managers and leaders in each community before the survey was tried. A principal concern was to minimise participant burden but also to permit each Local Government Council the opportunity to add their own locally-specific questions or propositions if they wished (for separate use in their submissions to the Queensland Government’s review). In keeping with the spirit and practice of reciprocity required for research with Indigenous Australians [40], upon completion of the survey, each community’s locally-specific survey results were returned to the Local Government Council and to the community at large via posters and pamphlets and in person by members of the research team [41].

### Data

So that the views of participants about the relative importance of each proposition could be gauged independently of the opinions of key community leaders, service providers and other stakeholders from which they were derived [15], participants were not informed of the definitions of ‘favourable’ or ‘unfavourable’ impacts. Thus blinded, and with the propositions ordered randomly in the survey (Table 1), for each of the 14 propositions, participants were asked to rate their agreement or disagreement using initial categories of: ‘strongly agree’; ‘agree’; ‘don’t know/unsure’; ‘disagree’; or ‘strongly disagree’. Since very few participants responded ‘don’t know/unsure’, this value was ignored. Binary variables were created for analyses (with values of ‘agree’ = 1 and ‘disagree’ = 0), by grouping the ‘agree’ with the ‘strongly agree’ ratings and the ‘disagree’ with the ‘strongly disagree’ ratings, respectively.

#### Demographic and other data

- *Sex and age group*: Males and females in age groups 18–24, 25–44, 45–64 and 65 years and older were included. These age groups anchor the experiences of community residents across historical periods. Prior to the mid-1960s there was no general access to alcohol for Indigenous Australians [42–45], a direct experience likely in the older age groups with older women in particular likely to be lifetime abstainers [46]. From the mid-1980s to 2002, alcohol became available in the communities surveyed, quite abruptly and with few effective limits, a direct experience of many (males and females) in the middle age groups [33]. Those in the youngest age group, born during the 1990s, will have experienced little other than restrictions or prohibition in their communities in their lifetimes.
- *Resident in the community for at least 6 years*: Although most residents of remote communities have spent much of their lives living there, those who were residents of the survey community before 2009, when the latest round of restrictions were implemented, were distinguished from those who were not.
- *Ethnicity*: Aboriginal and/or Torres Strait Islanders were distinguished from community residents of other ethnicities.
- *Current drinker*: Whether a current alcohol drinker or not assisted to assess any tendency in the patterns of responses to be influenced by alcohol users.

### Data analysis

#### Overview

Data analysis involved three steps. Firstly, the sample was described and the proportions agreeing with each proposition were summarised. Then, using logistic regressions, the influences of demographic and other factors on participant agreement with each proposition were assessed. Finally, using factor analyses, the relative importance of each proposition for participants in each of the two groups of ‘favourable’ and ‘unfavourable’ factors were assessed.

#### Description of the sample

**Fig. 1:**
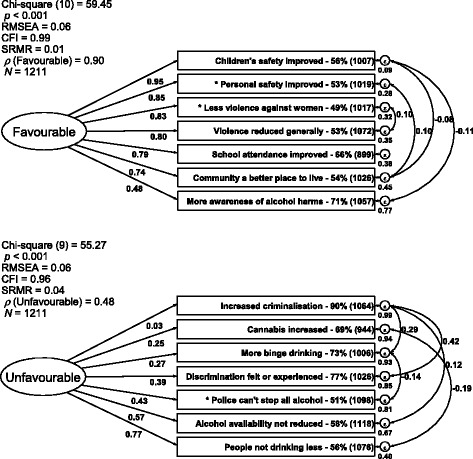
Measurement models of impacts of Alcohol Management Plans (AMPs) using confirmatory factor analysis (CFA) with structural equation modelling (SEM) analysing the tetrachoric correlation matrix for binary data from a survey of 1211 residents of 10 Aboriginal and Torres Strait Islander (Indigenous) communities conducted in Queensland (Australia) in 2014–15. The number of participants responding to each proposition is enclosed in *brackets* and the proportion agreeing about each proposition is included. * indicates where the stated proportion agreeing is not different from a theoretical proportion of 50%, i.e. no majority agreeing/disagreeing

Descriptive statistics summarised the sample characteristics. To compare the proportion agreeing with each proposition to a hypothesised proportion of 50% (i.e. no majority agreeing/disagreeing), one-sample proportion tests were used. These results are depicted in Table 1 (and also included in Fig. 1).

#### Binary logistic regressions

For each of the 14 propositions, multivariable, binary logistic regressions were conducted comparing those who ‘agreed’ with those who ‘disagreed’ (reference category) and examining the relative influence of demographic and other factors: sex, age group, resident in the community for 6 years and longer, ethnicity and current drinking status (see Table 2a and b). All regression modelling, using Stata 13© [47], estimated clustered robust standard errors to account for the clustering effects of data from 10 communities.

**Table 2:** Multivariable binary logistic regression models comparing characteristics of participants who agreed with each proposition about ‘favourable’ or ‘unfavourable’ impacts due to Alcohol Management Plans (AMPs) against those who disagreed (reference category) in a sample of 1211 residents of 10 Aboriginal and Torres Strait Islander (Indigenous) communities in a survey conducted in Queensland (Australia) in 2014–15

a) Participants who agreed with each proposition about ‘favourable’ impacts								
		Children safer	Personal safety improved	Violence against women reduced	Violence reduced generally	School attendance improved	Community a better place to live	More awareness of alcohol harms
Variable name		f1	f2	f3	f4	f5	f6	f7
Gender	Male	1.0 (0.9, 1.1)	1.1 (0.8, 1.5)	1.1 (0.8, 1.4)	1.0 (0.8, 1.2)	1.1 (0.9, 1.4)	1.0 (0.8, 1.4)	1.0 (0.7, 1.4)
Age group	18–24	0.5 (0.3, 1.1)	0.8 (0.4, 1.5)	0.7 (0.4, 1.2)	0.9 (0.5, 1.7)	0.6 (0.3, 1.0)*	0.7 (0.5, 1.2)	1.1 (0.5, 2.4)
	25–44	0.6 (0.3, 1.3)	0.5 (0.3, 1.1)	0.7 (0.4, 1.4)	0.9 (0.4, 1.7)	0.6 (0.3, 1.0)	0.5 (0.3, 0.9)*	0.8 (0.4, 1.7)
	45–64	0.6 (0.3, 1.2)	0.5 (0.3, 1.0)*	0.7 (0.4, 1.3)	0.8 (0.4, 1.4)	0.5 (0.3, 1.0)	0.6 (0.4, 0.8)*	0.9 (0.6, 1.5)
	65 and over†	1.0	1.0	1.0	1.0	1.0	1.0	1.0
Resident ≥ 6 years		1.2 (0.8, 1.6)	1.6 (1.1, 2.3)*	1.5 (1.0, 2.3)*	1.3 (0.9, 1.9)	1.0 (0.7, 1.3)	1.1 (0.8, 1.5)	1.4 (1.0, 1.8)*
Indigenous		1.1 (0.6, 2.1)	0.6 (0.4, 1.1)	1.1 (0.5, 2.5)	1.0 (0.5, 2.1)	1.6 (1.1, 2.5)*	0.7 (0.4, 1.2)	1.3 (0.6, 3.0)
Current drinker		1.3 (1.2, 1.5)*	1.0 (0.7, 1.5)	1.1 (0.8, 1.3)	1.0 (0.6, 1.6)	1.2 (0.9, 1.6)	0.9 (0.6, 1.3)	1.0 (0.8, 1.2)
b) Participants who agreed with each proposition about ‘unfavourable’ impacts								
		Increased criminalisation	Cannabis increased	Binge drinking increased	Discrimination felt or experienced	Police can’t stop all alcohol	Alcohol availability not reduced	People not drinking less
Variable name		u4	u1	u3	u6	u5	u7	u8
Gender	Male	1.0 (0.6, 1.6)	0.8 (0.6, 0.9)*	0.8 (0.6, 1.1)	1.0 (0.7, 1.3)	0.8 (0.6, 1.1)	0.9 (0.7, 1.2)	0.9 (0.6, 1.3)
Age group	18–24	0.5 (0.2, 1.9)	2.0 (1.2, 3.3)*	1.0 (0.4, 2.4)	1.8 (1.2, 2.9)*	1.3 (0.5, 3.3)	0.7 (0.4, 1.0)	0.8 (0.5, 1.3)
	25–44	0.8 (0.3, 2.3)	2.7 (1.6, 4.4)*	1.1 (0.5, 2.2)	1.9 (1.3, 2.7)*	1.7 (1.0, 2.9)*	0.6 (0.3, 1.1)	1.0 (0.6, 1.6)
	45–64	0.7 (0.2, 1.9)	2.4 (1.3, 4.4)*	0.9 (0.4, 2.0)	1.8 (1.1, 2.8)*	1.8 (1.0, 3.3)	0.6 (0.3, 1.2)	1.2 (0.7, 2.0)
	65 and over†	1.0	1.0	1.0	1.0	1.0	1.0	1.0
Resident ≥ 6 years		2.5 (1.3, 5.0)*	1.1 (0.8, 1.6)	1.5 (1.1, 2.1)*	1.2 (0.8, 1.6)	1.0 (0.7, 1.5)	0.8 (0.5, 1.1)	0.7 (0.4, 1.1)
Indigenous		1.5 (0.8, 3.0)	0.3 (0.1, 0.7)*	0.7 (0.5, 1.0)	1.2 (0.6, 2.5)	0.4 (0.2, 0.6)*	0.9 (0.5, 1.5)	0.5 (0.4, 0.7)*
Current drinker		0.9 (0.5, 1.5)	1.1 (0.8, 1.5)	0.7 (0.4, 1.0)*	0.9 (0.6, 1.3)	1.1 (0.7, 1.7)	1.0 (0.9, 1.2)	0.9 (0.6, 1.3)

*p < 0.05

† reference category

#### Confirmatory factor analysis (CFA) of survey data from 1211 community residents

To examine how propositions were correlated in participant responses and to assess the relative weight participants accorded to each, factor analyses were conducted. For these analyses, the concepts ‘favourable’ and ‘unfavourable’ were each treated as hypothetical latent constructs [48] reflected in the personal experiences community residents have had of the items proposed to them, although individuals’ experiences of AMPs will be multifarious. Confirmatory factor analysis (CFA) is required because the factors that underlie the responses to the two sets of seven items, i.e. the items comprising the ‘favourable’ and ‘unfavourable’ latent constructs, were pre-specified from analyses of interviews with key Indigenous community leaders service providers and stakeholders [48, 49]. CFA using structural equation modelling (SEM) in Stata 13© can model the fit of data to such theoretical constructs [50]. SEM reports factor loadings for each proposition and, because each item can have its own error variance, the covariation between items can also be assessed. Assisting to interpret the covariation of items, SEM also permits an estimate of reliability for each measurement model (CFA) [50]. These conceptual schemas are depicted in Fig. 1.

Tetrachoric correlations were first calculated (created as a positive, semi-definite correlation matrix using all available data to estimate correlations). Summary statistics data were then created for use in the analysis (see the tetrachoric correlations and the Stata 13© commands provided in the Additional file 1). Since CFA assumes that each latent variable summarises participants’ responses to the propositions [49, 50], the variances of the latent factors were each fixed at a value of 1, providing the standardized solutions presented in Fig. 1 and Table 3a and b. Co-efficients in the standardized solutions are interpreted as factor loadings [50], as in conventional factor analysis, and this permits straightforward comparisons between the co-efficients for each item.

**Table 3:** Standardised loadings for the CFA model depicted in Fig. 1 for seven propositions about ‘favourable’ and ‘unfavourable’ impacts of Alcohol Management Plans (AMPs) put to 1211 residents of 10 Aboriginal and Torres Strait Islander (Indigenous) communities in a survey conducted in Queensland (Australia) in 2014–15

Variable name	Propositions	Standardised loading ** *p* < 0.001 * *p* < 0.050
a) Propositions about ‘favourable’ impacts		
Loadings for variables		
f1	The AMP has helped make children safer in this community	0.95**
f2	The AMP has made people more safe in this community	0.85**
f3	The AMP has reduced violence against women in this community	0.83**
f4	Since the AMP, violence has gone down in this community	0.80**
f5	Since the AMP, school attendance has gone up in this community	0.79**
f6	The AMP has been good for this community and made it a better place to live	0.74**
f7	People are more aware of harmful effects of alcohol/drinking now (since the AMP)	0.48**
Variances		
error.f1		0.09
error.f2		0.28
error.f3		0.32
error.f4		0.35
error.f5		0.38
error.f6		0.45
error.f7		0.77
Favourable		1.00 (fixed)
Covariances		
error.f1 with error.f6		−0.08**
error.f1 with error.f7		−0.11**
error.f2 with error.f6		0.10**
error.f3 with error.f4		0.10**
b) Propositions about ‘unfavourable’ impacts		
Loadings for variables		
u4	The AMP has caused more people to get fined, criminal records and convictions	0.03
u1	There is more (not so much) gunjah being smoked in this community since the AMP	0.25**
u3	There is more “binge drinking” now than before the AMP	0.27**
u6	The AMP has discriminated against some people	0.39**
u5	Police can’t (can) enforce the AMP effectively and stop the alcohol coming in	0.43**
u7	The AMP has not (has) reduced the alcohol people can get in this community	0.57**
u8	The AMP has not (has) helped people change their drinking and they are (not) drinking less	0.77**
Variances		
error.u4		1.00
error.u1		0.90
error.u3		0.93
error.u6		0.85
error.u5		0.81
error.u7		0.67
error.u8		0.40
Unfavourable		1.00 (fixed)
Covariances		
error.u4 with error.u3		0.29**
error.u4 with error.u6		0.42**
error.u4 with error.u7		0.12**
error.u1 with error.u8		−0.19**
error.u3 with error.u5		−0.14**

## Results

### The sample

The sample of 1211 exceeded the designed sample size of 1160. It comprised 20% (=1211/5989) of the adult population resident in the 10 communities at the 2011 census. The sample proportions for the 588 (48%) males and 623 (52%) females in the sample, were similar to the proportions of males and females in the 2011 census (|z| = 1.21, *p* = 0.227, two sample test of proportions). Aboriginal and Torres Strait Islanders comprised 90% of the sample, also similar to census proportions of 92% (|z| = 1.82, *p* = 0.068). Most participants (86%) had lived in their community for at least 6 years. Current alcohol drinkers comprised 70% of the sample.

### Agreement with propositions about ‘favourable’ and ‘unfavourable’ impacts

As Table 1 (and Fig. 1) indicate, a narrow but statistically significant majority agreed with three of the seven propositions about ‘favourable’ impacts: 53% (*p* = 0.024) believed that violence had reduced in the community; 54% (*p* = 0.012) believed that the AMP was a good thing for their community making it a better place to live; and 56% (*p* < 0.001) thought that the AMP had made children safer. Participants were more equivocal that personal safety had improved (53% agreed, *p* = 0.097) and tended not to agree that there was less violence against women (49% agreed, *p* = 0.363). Agreement was clearer (66%, *p* < 0.001) that school attendance had improved and that people were more aware of the harmful effects of alcohol and drinking (71%, *p* < 0.001) since the AMP.

Agreement was quite a bit stronger about four of the ‘unfavourable’ impacts in particular (all *p* < 0.001): 69% agreed that cannabis use had increased; 73% agreed there was more binge drinking; 77% agreed that community people had experienced discrimination; and 90%, i.e. almost all participants, agreed that the AMP had caused more people to be fined and convicted and to receive criminal records. A smaller but statistically significant (*p* < 0.001) majority agreed that alcohol availability was not reduced (58%) and that people were not drinking less (56%). For the proposition that police could not enforce restrictions effectively and stop the alcohol coming into the community, participants held divided views (51% agreed, *p* = 0.365).

### Binary logistic regressions

Table 2a indicates that, across all ‘favourable’ impacts it was the longer term (≥ 6 years) residents who tended to be more likely to agree that personal safety had improved (OR = 1.6, 95% CI = 1.1, 2.3, *p* = 0.012), violence against women was reduced (OR = 1.5, 95% CI = 1.0, 2.3, *p* = 0.048) and that there was more awareness of alcohol harms in the community (OR = 1.4, 95% CI = 1.0, 1.8, *p* = 0.023). Table 2b indicates, however, that longer term residents also strongly agreed that binge drinking had increased (OR = 1.5, 95% CI = 1.1, 2.1, *p* = 0.023) and that there was increased criminalization because of AMPs (OR = 2.5, 95% CI = 1.3, 5.0, *p* = 0.009).

There was a moderate tendency for younger people to disagree about some of the ‘favourable’ impacts. For example compared with the oldest age group (aged 65 and over) those aged 25–44 were considerably less likely to agree that the community was a better place to live (OR = 0.5, 95% CI = 0.3, 0.9, *p* = 0.013). Similarly, those aged 18–24 years were less likely to agree that school attendance had improved (OR = 0.6, 95% CI = 0.3, 1.0, *p* = 0.040) (Table 2a). That discrimination was felt and experienced was a view held strongly among the younger people with views even stronger that cannabis use had increased following the AMP (see Table 2b).

Current drinkers held no particularly strong views about ‘favourable’ or ‘unfavourable’ impacts, except that they were less likely to agree that binge drinking had increased (OR = 0.7, 95% CI = 0.4, 1.0, *p* = 0.043) (Table 2b). Indigenous residents were more likely to agree that school attendance had improved (OR = 1.6, 95% CI = 1.1, 2.5, *p* = 0.025) (Table 2a) and less likely to agree that cannabis had increased (OR = 0.3, 95% CI = 0.1, 0.7, *p* = 0.003), that police couldn’t stop all alcohol coming in (OR = 0.4, 95% CI = 0.2, 0.6, *p* < 0.001) and that people were not drinking less (OR = 0.5, 95% CI = 0.4, 0.7, *p* < 0.001) (Table 2b).

### Confirmatory factor analysis

Following accepted reporting practice [51, 52], the standardized co-efficients from the SEM are depicted in Fig. 1 and Table 3a and b (also see Additional file 1).

#### ‘Favourable’ impacts

All pairs of tetrachoric correlation co-efficients between ‘favourable’ impacts were strong and statistically significant (*p* < 0.05) (see Additional file 1). ‘Favourable’ items are arranged in Fig. 1 and Table 3a in order of decreasing substantive strength of their loadings. In this model, six of the seven proposed ‘favourable’ impacts of AMPs on reducing violence (both generally and against women), improving children’s safety, school attendance and community amenity loaded significantly (*p* < 0.001) and quite strongly (absolute value of co-efficient |β| ≥ 0.5) [49] on the ‘favourable’ latent factor. The loading for increased awareness of alcohol harms was also statistically significant (*p* < 0.001) but weaker. The estimated reliability (*ρ* = 0.90) of this measurement model, stronger than an acceptable level (*ρ* = 0.70) [49], together with the weak co-variances (|β|~0.1), indicates that these items are pointing to a commonly agreed outcome of reduced violence and greater safety.

#### ‘Unfavourable’ impacts

The propositions about ‘unfavourable’ impacts are arranged in Fig. 1 and Table 3b in order of increasing substantive strength of their loadings. The low reliability (*ρ* = 0.48) suggests that the ‘unfavourable’ measurement model contains more than one natural grouping of items. The value for the covariance between the ‘discrimination’ and ‘increased criminalization’ items was very strong (0.42) (see Table 3b and Fig. 1). While their loadings on the single latent factor were comparatively weak (|β| ≤ 0.5), these items could be considered to represent a distinct sub-group of factors. This makes conceptual sense because of the strong community-wide feelings about these deleterious, social impacts (77 and 90% agreeing respectively) (Table 1 and Fig. 1). The moderate covariances among ‘people not drinking less’, ‘police can’t stop all alcohol’, ‘alcohol availability not reduced’ and ‘more binge drinking’ were also statistically significant (*p* < 0.05) demarcating this group of items related to alcohol availability and use from the discrimination and criminalization items (see Additional file 1). Finally, the ‘cannabis increased’ item covarying with ‘people not drinking less’, suggests an unmeasured group of items reflecting links between the availability and use of alcohol and cannabis.

#### Associations between ‘favourable’ and ‘unfavourable’ items

The primarily negative tetrachoric correlations between the individual ‘favourable’ and ‘unfavourable’ items (see Additional file 1) reflect the overall strong tendency for those who saw ‘favourable’ impacts to disagree that ‘unfavourable’ impacts had occurred, and vice versa. One exception to this pattern is the ‘increased criminalization’ item. For example, among the 566 of 1007 (=56%) participants who agreed that children’s safety had improved, the overwhelming majority also agreed that there had been increased criminalization (90%). Furthermore, more than two-thirds of the participants responding agreed that people had felt or experienced discrimination (69%). These were seen as major ‘unfavourable’ impacts, irrespective of views held about ‘favourable’ effects of AMPs.

### Summary

The results of this first survey of observations and experiences of adult residents in communities with an AMP in place in Queensland indicate that, by a narrow margin, they generally share the already-published views of long-term service providers, stakeholders and community leaders [15]. Participants recognized overall the ‘favourable’ impacts of AMPs on reducing violence, improving the safety of women and children, improved school attendance and community amenity, but with no overwhelming majority agreeing. These experiences are broadly consistent with the available objective evidence [18, 19] and other unpublished Queensland Government information [52] and commentary [14, 22, 53, 54]. Surrogate measures of alcohol-related injury progressively declined from 2002 after a period of poorly regulated alcohol availability beginning in the 1980s [18, 19]. Government statistics described reductions in interpersonal violence, but not in all communities [52].

Policy makers anticipated during the design phase that AMPs would have unintended consequences [26, 28]. Our survey shows that these have materialised in community residents’ experiences particularly in terms of the failure of AMPs to reduce alcohol availability and consumption. The social impacts of criminalization and discrimination were major concerns for a majority of participants irrespective of their views about the ‘favourable’ impacts. Clearly, these need to be addressed.

Longer term (≥ 6 years) residents were more likely to agree with propositions about ‘favourable’ impacts but also that there had been ‘unfavourable’ ones: increased criminalization and discrimination, changed drinking behaviours, and little impact on alcohol availability and its consumption. As the community populations age, perceptions of the ‘favourable’ impacts may become further eroded since, for example, younger people disagreed about the proposed ‘favourable’ impacts and also strongly agreed that discrimination was felt and experienced. Contrary to expectations, current drinkers held no particularly strong views about ‘favourable’ or ‘unfavourable’ impacts.

## Discussion

An absence of an overwhelming majority agreeing that AMPs had ‘favourable’ impacts does little to directly resolve the dilemma facing policy makers reviewing restrictions. Indigenous community leaders with the mandate (and the expectation) from their constituents to propose ways forward are also in an awkward position. Specifically, it is not clear that any ‘exit strategy’ that involves relaxation of restrictions with freer access to alcohol in itself would relieve the ‘unfavourable’ impacts identified, and a return to the very high rates of injury and death seen during the 1990s would be unconscionable. Indeed, that ‘favourable’ impacts have been experienced is a substantial achievement of major historical significance for Indigenous Australians which must be sustained. For considering ways forward out of the policy dilemma the substantial majorities agreeing about the identified ‘unfavourable’ impacts suggests that these should be addressed in ways that do not compromise the hard won ‘favourable’ achievements about which participants were more evenly divided in their views.

Firstly, the ‘unfavourable’ results where alcohol availability is seen as not reduced and with police not adequately resourced to enforce restrictions, is a difficult supply-side issue. It calls for Government to consider a new approach to controlling the supply of alcohol in the communities. This could be achieved with strategies tried in other Australian jurisdictions to control access to alcohol at the point of sale [22]. The liquor industry needs to be appropriately engaged by Government, with a particular focus on the ‘catchment’ licensed premises. Adequate resources are needed for the enforcement originally required or implied by the AMP legislation [23]. Indigenous community leadership could advocate for such strategies while also fostering local governance mechanisms and surveillance aimed at making it more difficult for illicit alcohol to enter the community. The failure to reduce alcohol availability with effective enforcement was linked with people not changing their drinking patterns and with more binge drinking seen. Demand-side issues such as binge drinking could be addressed with scaled-up treatment and diversion options provided by government and community-controlled services [55] in the affected communities, particularly with at-risk groups within the population. Any chronically addicted drinkers comprises one such target group, of course [56–59]. The younger age groups represent another. The results further suggest that scaled-up treatment and diversion options would have collateral benefits if they had a focus on addressing illicit drugs and their substitution with alcohol.

Justifying survey participants’ near-ubiquitous concerns about increased criminalization, publicly available Queensland Government information [60, 61] indicates that, up to the 30th of June 2014, a total of 6961 distinct persons had been convicted of 15,511 charges for breaching S168B and/or 168C of the Liquor Act. Over 100 were incarcerated. Almost all those charged and convicted were Indigenous residents of AMP communities. This means that possibly 70% of adults had accrued at least one conviction by mid-2014 when this survey was conducted. This issue underpinned the distress expressed by many participants in all communities, and so deserves priority attention.

Finally, the legal designation of AMPs as a ‘special measure’ under Australia’s Racial Discrimination Act (1974) [23, 62] does little to assist a nuanced understanding of how Indigenous residents of AMP communities, and of the ‘catchment’ towns nearby, can feel discrimination in their everyday lives while going about routine activities including purchasing alcohol at liquor outlets. Uninformed sales personnel reportedly cause offence by applying ‘harm minimisation’ conditions inappropriately. Education for liquor retailers in ‘catchment’ licensed premises (and their employees) would increase their cultural competence and reduce the scope for discrimination to occur. Both the Queensland Government and the affected communities and their advocates have a role to play in such a strategy.

### Limitations and strengths of the study

An unavoidable weakness of this study is that neither the participants nor the communities were randomly selected, limiting the capacity to generalize the results. However, the large sample size represents a substantial proportion (20%) of the adult resident population. The sample size was large enough to provide sufficient study power to reliably detect differences between groups with opinion closely divided. A smaller sample size would have produced more equivocal results, providing no clear policy direction.

Communities were self-selecting, i.e. where permission to conduct the survey was provided by the Local Government Council, further demonstrating the impracticability of random sampling. The 10 communities nonetheless represented two-thirds of the communities located in the 15 restricted areas with AMPs in place.

A considerable strength of this study is that it was guided by NHMRC principles of reciprocity and respectful community engagement in a very challenging and controversial topic area. Because of the emotive nature of the issue in AMP communities, data collection in the way described was, arguably, the only practical way to conduct such a study. Moreover, it is unlikely that resources will be available for any similar survey in the near future and the feasibility of any future study would face similar practical challenges.

The weak binary measures used in the study could be improved with more research to develop more sensitive indicators. However, as the analysis stands, it provides decision-makers with some practical options for policy change that are backed by the first robust evidence of its kind in the literature.

## Conclusion

The dilemma facing policy makers reviewing AMPS would appear, on its face, to be insurmountable. With any risk of compromising community safety unacceptable, the evidence reported here suggests that alcohol restrictions should be maintained more or less in their current form for the foreseeable future in Queensland’s Indigenous communities. The present circumstances wherein AMPs are subject to review provide an important opportunity for a thorough and respectful consultation process which can target the issues of concern for communities identified, namely: reconciliation of the issues of criminalization and discrimination, addressing illicit alcohol and the provision of treatment and diversion services together with cultural awareness education for liquor retailers. These particular areas are largely the mandate of Government. The Local Government Councils in AMP communities, although lacking in resources, would likely play a constructive supporting role in addressing these issues provided there is democratic and appropriate consultation and engagement.

The results also suggest a role for research and evaluation. Deliberative democracy approaches [63] to develop suites of remedial evidence-based strategies, combined with community-inspired ideas would be appropriate to address the past lack of consultation with affected communities. Such approaches, increasingly used in considering complex ethical issues and policies in health systems research, would address in a pragmatic way calls for genuine government collaboration and engagement with Indigenous communities [22, 53, 54].

## Additional file

Additional file 1: Tetrachoric correlation co-efficients and Stata 13 commands for summary statistics data. (DOCX 50 kb)
