# Supplementary material for: Alcohol management plans in Aboriginal and Torres Strait Islander (Indigenous) Australian communities in Queensland: community residents have experienced favourable impacts but also suffered unfavourable ones
Source: BMC Public Health. 2017 Jan 10;17:55. doi: 10.1186/s12889-016-3995-8 (PMC5223386; doi:10.1186/s12889-016-3995-8)
Supplement: Additional file 1: — Tetrachoric correlation co-efficients and Stata 13 commands for summary statistics data. (DOCX 50 kb) [file 12889_2016_3995_MOESM1_ESM.docx]

ADDITIONAL FILE 1

| Tetrachoric correlation co-efficients (* P<0.05) | Variable names (* P<0.05) | | | | | | | | | | | | | | |
| --- | --- | --- | --- | --- | --- | --- | --- | --- | --- | --- | --- | --- | --- | --- | --- |
| Latent variables |  | Favourable | | | | | | | Unfavourable | | | | | | |
| Indicator labels |  | f1 | f2 | f3 | f4 | f5 | f6 | f7 | u4 | u1 | u3 | u6 | u5 | u7 | u8 |
| *Children’s safety improved* | f1 | 1.0000 |  |  |  |  |  |  |  |  |  |  |  |  |  |
| *Personal safety improved* | f2 | 0.8121* | 1.0000 |  |  |  |  |  |  |  |  |  |  |  |  |
| *Less violence against women* | f3 | 0.7935* | 0.6793* | 1.0000 |  |  |  |  |  |  |  |  |  |  |  |
| *Violence reduced generally* | f4 | 0.7627* | 0.7227* | 0.7690* | 1.0000 |  |  |  |  |  |  |  |  |  |  |
| *School attendance improved* | f5 | 0.7507* | 0.6741* | 0.6316* | 0.6348* | 1.0000 |  |  |  |  |  |  |  |  |  |
| *Community a better place to live* | f6 | 0.6290* | 0.7339* | 0.5971* | 0.5920* | 0.6086* | 1.0000 |  |  |  |  |  |  |  |  |
| *More awareness of alcohol harms* | f7 | 0.3509* | 0.3907* | 0.4092* | 0.4102* | 0.3795* | 0.3414* | 1.0000 |  |  |  |  |  |  |  |
| *Increased criminalisation* | u4 | 0.0804 | -0.0573 | 0.0551 | 0.0777 | 0.1196 | -0.1339 | 0.1451* | 1.0000 |  |  |  |  |  |  |
| *Cannabis increased* | u1 | -0.2064* | -0.1506* | -0.1656* | -0.2310* | -0.3131* | -0.2599* | 0.0067 | -0.0385 | 1.0000 |  |  |  |  |  |
| *More “binge drinking”* | u3 | -0.1515* | -0.1702* | 0.0061 | -0.0568 | -0.1235* | -0.1231* | 0.0168 | 0.3186* | 0.0309 | 1.0000 |  |  |  |  |
| *Discrimination felt or experienced* | u6 | -0.2958* | -0.3460* | -0.2258* | -0.2397* | -0.2660* | -0.4095* | 0.0261 | 0.4488* | 0.1014 | 0.1593* | 1.0000 |  |  |  |
| *Police can’t stop all alcohol* | u5 | -0.3079* | -0.3903* | -0.1993* | -0.2033* | -0.2769* | -0.3949* | -0.1091* | 0.0720 | 0.1986* | -0.0106 | 0.2486* | 1.0000 |  |  |
| *Alcohol availability not reduced* | u7 | -0.4536* | -0.5031* | -0.4289* | -0.4163* | -0.3698* | -0.4563* | -0.3791* | 0.1036 | 0.0742 | 0.1356* | 0.1708* | 0.2456* | 1.0000 |  |
| *People not drinking less* | u8 | -0.6154* | -0.6973* | -0.5568* | -0.5806* | -0.5695* | -0.6057* | -0.3391* | 0.0318 | 0.0027 | 0.2157* | 0.2905* | 0.2920* | 0.4772* | 1.0000 |

*Tetrachoric correlation co-efficients*

*Stata commands for summary statistics data*

Summary statistics data for use in the analyses can be created using the following Stata 13© commands:

**ssd init** f1 f2 f3 f4 f5 f6 f7 u4 u1 u3 u6 u5 u7 u8

**ssd set obs** 1211

**ssd set correlations** 1.0000 \ 0.8121 1.0000 \ 0.7935 0.6793 1.0000 \ 0.7627 0.7227 0.7690 1.0000 \ 0.7507 0.6741 0.6316 0.6348 1.0000 \ 0.6290 0.7339 0.5971 0.5920 0.6086 1.0000 \ 0.3509 0.3907 0.4092 0.4102 0.3795 0.3414 1.0000 \ 0.0804 -0.0573 0.0551 0.0777 0.1196 -0.1339 0.1451 1.0000 \ -0.2064 -0.1506 -0.1656 -0.2310 -0.3131 -0.2599 0.0067 -0.0385 1.0000 \ -0.1515 -0.1702 0.0061 -0.0568 -0.1235 -0.1231 0.0168 0.3186 0.0309 1.0000 \ -0.2958 -0.3460 -0.2258 -0.2397 -0.2660 -0.4095 0.0261 0.4488 0.1014 0.1593 1.0000 \ -0.3079 -0.3903 -0.1993 -0.2033 -0.2769 -0.3949 -0.1091 0.0720 0.1986 -0.0106 0.2486 1.0000 \ -0.4536 -0.5031 -0.4289 -0.4163 -0.3698 -0.4563 -0.3791 0.1036 0.0742 0.1356 0.1708 0.2456 1.0000 \ -0.6154 -0.6973 -0.5568 -0.5806 -0.5695 -0.6057 -0.3391 0.0318 0.0027 0.2157 0.2905 0.2920 0.4772 1.0000

*Structural Equation Modelling*

*‘Favourable impacts’*

In the initial model (not shown), all items loaded significantly on the single ‘favourable’ dimension. The fit of the initial model was very poor (χ2(14)=396.85, P<001; root mean square error of approximation (RMSEA)=0.15 greater than the acceptable value of 0.08. The standardised root mean squared residual (SRMR)=0.03 was less than the accepted value of 0.05; and comparative fit index (CFI)=0.94 approached an acceptable value of 0.95^1-3^. The modification indices suggested several pairs of correlated variables. These made conceptual sense as the factors ‘more awareness of alcohol’s harms’ and ‘community a better place to live’ are likely to be linked with ‘improved personal safety’ and ‘children’s safety’ in particular. Linking ‘less violence against women’ and ‘reduced violence generally’ also made conceptual sense. The fit of the modified model, as indicated by a large, significant chi-square value, was not ideal: χ2(10)=59.45, P<001. However, the goodness of fit measures were acceptable: RMSEA=0.06 and SRMR=0.01 both less than the acceptable values of 0.08 and 0.05 respectively; and CFI=0.99 higher than 0.95. The reliability of the measurement model for ‘favourable’ impacts was 0.90, considerably greater than an acceptable level of 0.70.

*‘Unfavourable’ impacts*

The fit of this model was also initially very poor (χ2(14)=506.67, P<001; RMSEA=0.17; SRMR=0.10 and CFI=0.57). The modification indices suggested several pairs of correlated variables for the model. Although their conceptual sense was not clear, when these correlations were included in the model, the goodness of fit measures improved substantially, although still not ideal (χ2(9)=55.27, P<001; RMSEA=0.06; SRMR=0.04; CFI=0.96). The reliability of the measurement model for ‘unfavourable’ impacts was just 0.48, considerably less than 0.70.

References cited

1. Acock AC. Discovering structural equation modeling using Stata. Revised edition. College Station, Texas: Stata Press; 2013.

2. Hoyle RH. Handbook of Structural Equation Modeling. New York: Guilford Publications; 2014.

3. Hu Lt, Bentler PM. Cutoff criteria for fit indexes in covariance structure analysis: Conventional criteria versus new alternatives. Struct Equ Modeling 1999;6:1-55.
